# Statistically significant results from low-power analyses: A comedy of errors

**DOI:** 10.1016/j.gloepi.2026.100250

**Published:** 2026-01-16

**Authors:** Cyril Jaksic, Thomas Perneger, Christophe Combescure

**Affiliations:** Clinical Research Centre, University Hospitals of Geneva, Geneva, Switzerland

**Keywords:** Statistical Bias, Statistical significance, Low power

## Abstract

**Background:**

When low-power analyses yield statistically significant results, they likely overestimate the true effect. Although sample estimates are symmetrically distributed around the true value, those that are by chance very high are more likely to achieve statistical significance. The bias induced by the significance filter increases as power decreases. Here we sought to quantify the estimation bias associated with low power and to contrast it with the type M error, which assesses the same phenomenon from a different perspective.

**Methods:**

We used simulations to quantify estimation bias in relation to power among statistically significant results. We computed the type M error, relative bias (ratio of the estimated mean differences and the true value), and proportions of results with various levels of over- and under-estimation.

**Results:**

For a medium effect size (Cohen's d of 0.5), overestimation of the mean difference was moderate at high power (≥80%): relative bias was <1.13, about 65% of estimates were roughly accurate (between 0.75 and 1.25 of the true value), and sign errors were virtually absent. In contrast, at low power (<30%), overestimation was strong (relative bias >1.78), and almost no estimates were roughly accurate. Sign errors became noticeably prevalent only at very low levels of power (<10%). In all situations, the relative bias had a lower magnitude than the type M error.

**Conclusion:**

Low-power statistically significant results may consist entirely of magnitude errors, sign errors, and type 1 errors with high risk of strong overestimation (double effect). Readers should beware positive results from low-power analyses.

## Highlights

• Among statistically significant results, the true effect is globally overestimated.
• This bias increases as the statistical power decreases.
• In analyses with a power of 25%, the positive significant effects are twice the true effect.
• Readers of research reports should beware positive results of low-power analyses.
• Publication decision should be based on the quality of the study, not the results.

**Table:** 

Key findings - Considering only statistically significant results is a source of bias in the estimation of an effect; the magnitude of this bias and its relationship with the power of the analyses are considered using a simulation study. What this adds to what was known? - This bias is function of the statistical power, and is particularly important in low power analyses (e.g., interim analyses, secondary analyses, small sample size studies). - The relative bias has advantageous properties for interpretation compared to the commonly cited metric type M error. What is the implication and what should change now? - Readers should not mostly consider significant results and critically assess results in situations where low statistical power was likely. - Researchers should report all conducted analyses, regardless of their statistical significance - Editors should publish all submitted results, regardless of their statistical significance

## Introduction

In public health and clinical research, the evidence is mainly produced by testing hypotheses on data. Typically, in a clinical trial one may hypothesize that two drugs have different effectiveness on a health outcome. Once data are collected, a statistical test can either provide a statistically significant result or not. The statistical significance is usually assessed from a *p*-value. A low p-value indicates that study data are poorly compatible with the null hypothesis being that the two drugs have actually the same effectiveness. Commonly, *p*-values below 0.05 lead to the rejection of the null hypothesis and to the conclusion that an effect exists; the result is then said to be statistically significant. A non-statistically significant result means that the test failed to reject the null hypothesis: the lack of statistical significance is not the evidence of the absence of effect [1]. Beyond merely detecting an effect through a statistical test, estimating its magnitude (for example, a mean difference or a risk ratio) is essential to interpret the clinical relevance of the findings [2]. The magnitude of the estimated effect and the statistical significance are related: the larger the observed effect—whether negative or positive—the less compatible it tends to be with the null hypothesis and the more likely it is to be statistically significant. Therefore, the set of statistically significant estimates is not representative of all estimates. Statistical significance also depends on the sample size: for the same observed effect (and observed standard deviation), the *p*-value decreases as the sample size increases.

The ability of a study to detect an effect when it truly exists is one of its fundamental characteristics. Through sample size calculation, most phase 3 clinical trials are designed to achieve a statistically significant result on the primary outcome with a statistical power of 0.8 or 0.9. In other words, they aim for an 80% or 90% probability of rejecting the null hypothesis if the treatment truly has the assumed level of efficacy—this probability being what defines statistical power. Nevertheless, low-power analyses abound in clinical research [3], [4]: analyses of secondary outcomes or safety events in clinical trials [5], subgroup analyses [6], interim analyses [7], phase 2 trials [8], trials that were terminated due to recruitment problems [9], and various types of observational or hypothesis-generating studies. Since the distribution of effect estimates depends on the sample size, the likelihood of obtaining an estimate far from the true effect is higher when the power is low than when it is high. Moreover, as power decreases, the proportion of statistically significant results diminishes, and these results become increasingly poorly representative of the set of all estimates. Thus, low power greatly amplifies the poor representativeness of statistically significant estimates. Low power also increases the chance of statistically significant results to have the wrong sign. For example, when the true mean difference (delta) is positive, low-powered studies are more likely to produce a statistically significant estimate that is negative. This is called a type S (sign) error [10]. Unlike a type 1 error, which incorrectly rejects the null hypothesis (i.e. wrongly concludes an effect exists), a type S error correctly rejects the null hypothesis but with an estimated effect in the wrong direction.

In clinical research, statistically significant results are more likely to be published [11]. This can be explained by several factors including the preference for journals to publish studies with statistically significant results and researchers, discouraged by such practices, not attempting to publish non-significant results [12]. Consequently, the published results are not representative of all research findings and may give readers a distorted picture of a drug's effect. The magnitude of this distortion on the effect size (a standardized estimate of the effect) and its relationship with the power have been examined using a metric called the *type M* (magnitude) error [10], [13]. This metric quantifies the inconsistency between the true and the estimated effect size in the extreme situation where only statistically significant results are considered and non-statistically results are discarded, that is the results are filtered on their statistical significance. The type M error has been shown to be close to 1 (i.e. no distortion) for a power close to 100% (almost all estimated effect sizes are statistically significant) and to increase substantially when the statistical power decreases.

Technically, the type M error is the mean of the absolute values of the statistically significant effect sizes divided by the true effect size [10], [13]. It does not correspond to the definition of a bias which is the difference between the expected value of the estimate and the value of the true value. The literature suggests that the type M error represents the extent to which the effect sizes of statistically significant results overestimate the true value, hence being also coined the *exaggeration ratio*. However, the exaggeration can be misinterpreted as being only due to the *significance filter* [14]. For example, for a true Cohen's d = 0.2 under a two-sided *t-*test (alpha = 0.05) and a power of 10%, the type M error is around 3.5. It would be a mistake to assume that filtering on statistically significant results in a 3.5-fold exaggeration of the expected effect size because, in the absence of a filter on statistical significance, the same computation with the same power leads to a value of 1.4, not 1. Another limitation of the type M error is that it is defined on standardized effects, whereas clinical research more commonly interprets raw metrics such as mean differences. The magnitude and practical implications of bias in raw units differ from those in standardized units. For these reasons, we propose a more direct, intuitive and interpretable metric that directly quantifies the bias specifically attributable to significance filtering in the natural units of interest.

Here we quantify the bias in the estimation of a difference in means due to the significance filter with varying statistical power using simulations, and compare the magnitude of that bias to the type M error. We also estimate the probability of type S errors as a function of statistical power. Several conditions are tested in order to understand the behavior of this in situations encountered in clinical research (one-sided statistical test, unbalanced group sizes, unequal variances, alpha risk lower than 0.05, Welch's *t*-test or z-test rather than Student's t-test).

## Methods

### Setup

We conducted a simulation study based on the replication of a superiority randomized trial that compares a new treatment to usual care. The efficacy of the new treatment is assessed by a difference in means (e.g., mean values of diastolic blood pressure (DBP) in patients with hypertension).

### Simulations

Briefly, data (sample means and standard deviations) of a large number of studies were randomly generated for statistical powers ranging from 6 to 95% under a true mean difference of 0.2, 0.5 and 0.8. The true pooled standard deviation was set to 1 such that the true values of Cohen's d were equal to the mean difference and correspond to effect sizes commonly deemed low, medium and high respectively [15]. The distribution of the statistically significant effect was thus assessed.

In details, for each generated study, the size of the two samples (for instance representing patients on usual care and on experimental treatment) was determined by the power calculation with a normally distributed outcome variable. In the power calculation, the mean difference between the populations and the standard deviation in each group were set to the true values. There is no loss of generality by considering a true pooled standard deviation of 1. For example, an outcome variable might be a difference in DBP (ΔDBP) between baseline and follow-up. If we assume a standard deviation of 10 mmHg for ΔDBP, the investigated scenarios correspond to true mean differences in ΔDBP of 2, 5, or 8 mmHg between the interventions.

### Data generation

When the experimental treatment is actually effective, the data were randomly generated as follows:

a) Set a true mean difference of 0.2, 0.5 or 0.8 and determine the true standard deviations that lead to a pooled standard deviation of 1 and thus a true Cohen's d of 0.2, 0.5 or 0.8 (see Appendix 1).
b) Compute the sample size needed to achieve a given level of power by using the classical formula for the comparison of means shown in Appendix 1 (e.g., with effect size 0.2, two-sided type 1 error 0.05, and power 80%, the required sample size is 2 × 394, for power 50% the required sample size is 2 × 193, and for power 0.20 the required sample size is 2 × 63).
c) Determine the parameters of the distributions of sample means (normal distribution) and standard deviations (chi-squared distribution) corresponding to the sample size computed in a). The distributions are shown in Appendix 1.
d) For each study arm, randomly generate a sample mean and a standard deviation from the distributions determined in c).
e) Repeat step d) 500,000/power times. Because our analyses solely rely on significant results, this procedure leads to an expected number of statistically significant results of 500,000. This ensures precise estimations of the bias.

The steps b) to e) were repeated for statistical powers ranging from 6 to 95%. This process was used to generate data under three conditions: 1) true standard deviation equal and balanced groups (base case scenario), 2) true standard deviation equal and unbalanced groups with an allocation ratio 1:4, 3) balanced groups and unequal true standard deviations with a ratio 1:2. The sample size calculation in step b) takes into account the imbalance between groups. To obtain the desired true Cohen's d, the true standard deviation was set to 1 in the condition with equal standard deviations, and to √(5/3) and 2√(5/3) in the condition with unequal standard deviations.

### Analysis of generated data

We analyzed the randomly generated data of each study using several statistical procedures. The default procedure consisted of a two-sided *t*-test assuming equal variances and with an alpha threshold of 0.05. The other procedures with an alpha threshold of 0.05 were: one-sided t-test, Welch's t-test, z-test. The two-sided t-test with an alpha threshold of 0.01 was also investigated. The scenarios that we investigated are a combination of the condition under which data are generated and of the statistical procedure that was applied to determine the statistical significance. They are summarized in Table 1.

**Table 1:** Investigated scenarios in the simulations.

	Conditions of data generation		Statistical procedure to identify significant results		
Scenario	Standard deviations (SD)	Groups size	Statistical test	Alpha threshold	One/two-sided
1	equal	balanced	t-test	0.05	two-sided
2	unequal (1:2)	balanced	t-test	0.05	two-sided
3	equal	unbalanced (1:4)	t-test	0.05	two-sided
4	equal	balanced	z-test	0.05	two-sided
5	equal	balanced	Welch's t-test	0.05	two-sided
6	equal	balanced	t-test	0.05	one-sided
7	equal	balanced	t-test	0.01	two-sided

For each procedure, the following metrics were calculated solely on the statistically significant results:

- Type S error rate: this corresponds to the proportion of estimates that have the wrong sign (i.e. negative estimate of the mean difference while the true mean difference is positive)
- Type M error: the ratio between the mean of absolute Cohen's d estimates and the true Cohen's d.
- Relative bias: the ratio between the mean estimated deltas (means differences) and the true delta. Here, the signs of the deltas are preserved in order to reflect the actual bias in estimation.
- Relative positive (respectively negative) bias: the ratio between the mean of positive (respectively negative) deltas and the true delta. The positive bias was also categorized into what we deemed moderate underestimation (bias between 0 and 0.75), roughly correct estimations (0.75 to 1.25), moderate overestimation (1.25 to 2), and strong overestimation (>2).

We describe the above metrics for each of the effect size (0.2, 0.5 and 0.8) as a function of power.

### Mixture of alternate and null hypotheses

Because in real life one does not know the true effect, we considered the situation where multiple tests are performed (e.g., multiple clinical trials of various treatments), some of them with a true effect in favour of the experimental treatment, and some without effect. In the latter case, a result can be statistically significant solely by chance (type 1 error). To produce effectiveness estimates when there is no effect in reality, we proceeded as described above (scenario 1 in Table 1), except that both samples were drawn from the same population (i.e. same mean and same standard deviation), that corresponds to a true mean difference of 0. The statistically significant results were classified as type 1 error with correct sign (with respect to the true mean difference), and type 1 error of wrong sign. The distribution of statistically significant estimates was obtained for all results. The proportion of estimates that were generated from a true mean difference of 0 was 25, 50 and 75%. The other estimates were generated from a true mean difference of 0.5. The standard deviation was set to 1 so that corresponding Cohen's d were 0 and 0.5 respectively.

## Results

Unless specified otherwise, all results are reported for a comparison of means between two independent groups of same size and same variance, using a two-sided Student *t*-test, with a threshold alpha of 0.05 (scenario 1 in Table 1), and with a true effect size of Cohen's d = 0.5 (considered as medium).

**Fig. 1:**
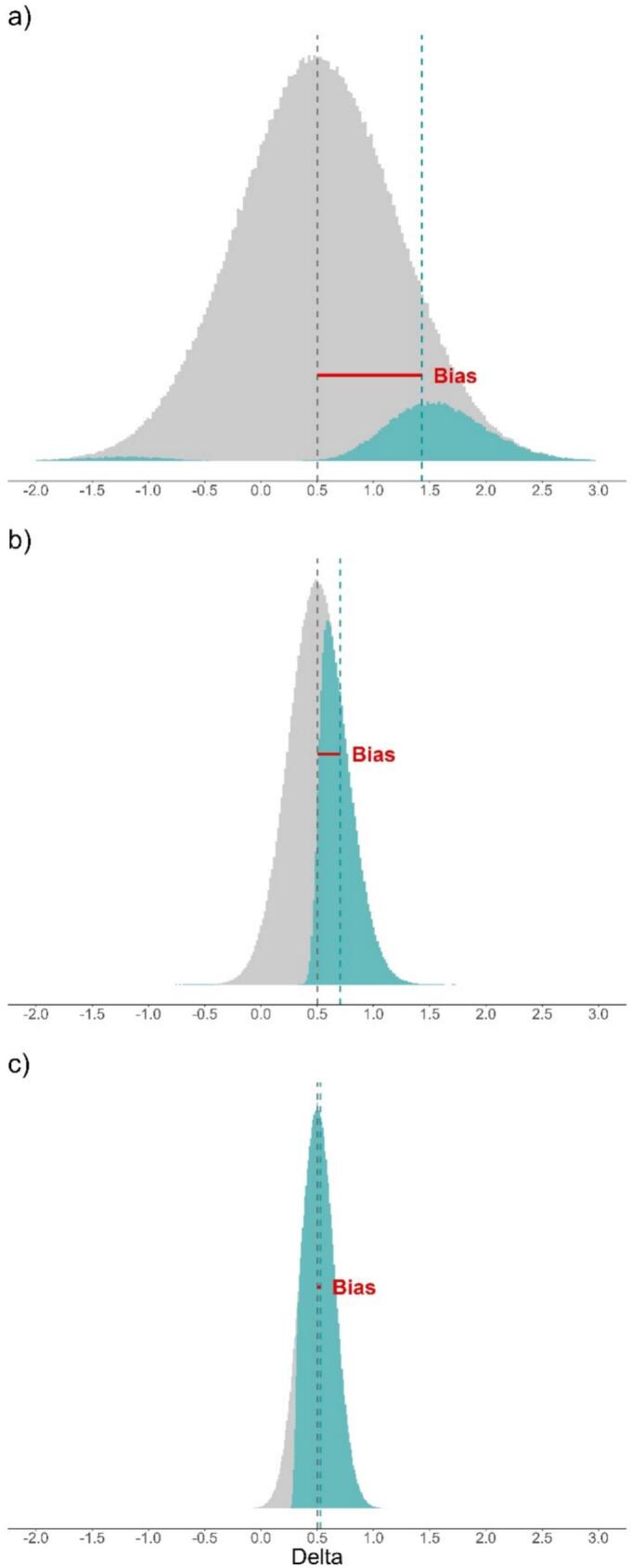
Distributions of all deltas, from a population with a true delta of 0.5 and standard deviation in both groups of 1 (Cohen's d = 0.5), with the statistically significant deltas in green under a power of a) 10%, b) 50% and c) 90%. (For interpretation of the references to colour in this figure legend, the reader is referred to the web version of this article.)

As per Table 2, type S error rate only reaches values above 1% with the statistical powers below 20%, and is virtually non-existent for powers above 70%. Both type M error and the relative bias are close to 1 with high statistical power, and increase as the power decreases. The relative bias was around 1.15 for a power of 80%, that is commonly targeted in clinical trials, and showed a strong bias for a power of 10%. The relative bias was systematically lower than the type M error but the contrast was sensitive only for low powers. Examples of distributions of effectiveness estimates (mean differences) under various levels of power are shown in Fig. 1. With a power of 10% (Fig. 1a), the distributions of significant estimates resemble bimodal bell-shaped distributions. The left part corresponds to negative significant estimates and the right part to positive significant estimates. The difference between the mean of all statistically significant results and the true value represents the absolute bias on the effectiveness estimates induced by significance filtering. With higher powers (50% in Fig. 1b and 90% in Fig. 1c), negative significant estimates are rare and the distribution of positive significant estimates approaches a truncated distribution.

**Table 2:** Type S and M error, and the relative bias considered all significant results for different statistical power, with a true effect size of Cohen's d = 0.5.

Power	Type S error rate (%)	Type M error	Relative bias (delta)
0.1	5.5	4.74	2.86
0.2	0.5	2.35	2.12
0.3	0.1	1.88	1.78
0.4	<0.1	1.60	1.55
0.5	<0.1	1.44	1.41
0.6	<0.1	1.31	1.29
0.7	<0.1	1.21	1.20
0.8	0	1.14	1.13
0.9	0	1.06	1.06
0.95	0	1.03	1.03

When the power was less than 30%, less than 5% of the positive significant estimates were roughly correct (i.e. that did not deviate from the true value by more than 25%) (Table 3). While a high power prevented strong overestimations, the risk that a positive significant estimate be twice as high as the true value exceeded 10% for power less than 40%. With a very low power (10%), a strong overestimation was almost systematic (92.3%). As power decreased, not only did the type S error rate increase, but the magnitude of significant negative estimates also increased as well.

**Table 3:** Relative bias of statistically significant deltas when only considering the positive or the negative deltas, as a function of power. In addition, the proportions of categories (moderate underestimation, roughly correct estimate, moderate overestimation and strong overestimation) of relative bias for positive deltas are reported. The results are shown for a Cohen's d of 0.5.

		Positive deltas				
Power	Relative bias in negative deltas	Relative bias	Moderate underestimation (RB 0–0.75) %	Roughly correct estimate (RB 0.75–1.25) %	Moderate overestimation (RB 1.25–2) %	Strong overestimation (RB > 2) %
0.1	−2.54	3.18	<0.1	0.4	7.2	92.3
0.2	−1.73	2.14	<0.1	0.6	42.1	57.3
0.3	−1.43	1.78	0	4.0	71.2	24.8
0.4	−1.21	1.55	<0.1	18.3	71.2	10.5
0.5	−1.06	1.41	<0.1	36.7	58.3	5.0
0.6	−0.96	1.29	<0.1	51.7	46.1	2.1
0.7	−1.22	1.20	0.8	61.0	37.3	0.9
0.8	–	1.13	5.0	64.6	30.1	0.3
0.9	–	1.06	11.9	65.1	23.0	<0.1
0.95	–	1.03	13.9	66.7	19.3	<0.1

Similar relationships between the type S/M errors, relative bias and power were obtained for Cohen's d values of 0.2 and 0.8 but with different magnitudes (see Appendices 2 and 3). However, at the same level of power, the type S error rate and the type M error increased with the true effect size but the relative bias decreased. The contrast is particularly striking for a very low power (10%): the type M error ranged from 3.64 for Cohen's d = 0.2 to 11.16 for Cohen's d = 0.8 and the relative bias ranged from 3.22 to 2.13. In more technical terms, this contrast is explained by the fact that in order to maintain a given power, varying Cohen's d requires a change in the sample size; increasing Cohen's d require smaller sample size. In turn, smaller sample sizes lead to sample standard deviations of greater amplitude with noticeably more extreme values (high values or very close to zero). When a sample standard deviation is close to zero, it increases the chance for a statistically significant result. As a result, a very small sample delta could be statistically significant with a very small sample standard deviation. These values contribute to reducing the relative bias. On the other hand, Cohen's d value is computed using the standard deviation (as denominator), so an extreme standard deviation that nears zero is not only likely to generate a statistically significant result but also to lead to an extremely high value of Cohen's d. Since type M error is calculated based on Cohen's d, maintaining a constant power while increasing Cohen's d will increase type M error. The joint distribution of sample standard deviation and delta or Cohen's d under different sample size is shown in Appendix 4.

**Fig. 2:**
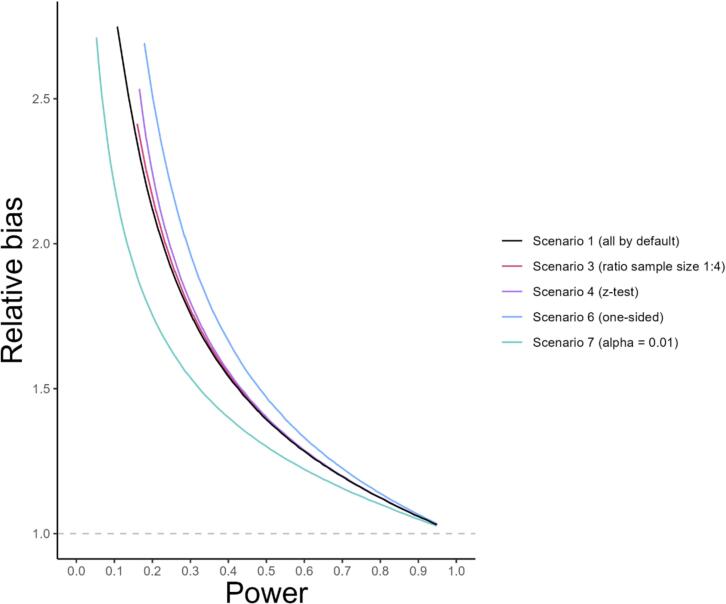
Relative bias as function of statistical power for different conditions. Only simulations with sample size in both compared groups of five or greater are shown. The scenario 1 (default options, in black) corresponds to a two-sided Student t-test with a threshold alpha of 0.05, comparing two groups of equal sample sizes and equal variances. Each modified parameter was changed while keeping all the other ones as per their default setting. The ratio of sample sizes in both groups was changed to 1:4 (scenario 3, red), the test statistic was changed to the z-test (scenario 4, purple), the test was changed to a one-sided test (scenario 6, blue), and the alpha threshold was changed to 0.01 (scenario 7, green). (For interpretation of the references to colour in this figure legend, the reader is referred to the web version of this article.)

Fig. 2 represents the relative bias as a function of power for different scenarios (see Table 1). The relative bias was significantly higher when using a one-sided Student *t*-test (scenario 6) instead of a two-sided test at same power (scenario 1) and it was significantly lower when using a significance level of 0.01 (scenario 7) instead of 0.05. When the ratio of the sample size differed from 1:1, using a 1:4 ratio instead (scenario 3), the relative bias only marginally increased. The same happened when using a z-test instead of the t-test (scenario 4). Replacing the equal-variance Student t-test with Welch's t-test (scenario 5), or varying the ratio of the two group's standard deviations (scenario 2) only produced negligeable changes in relative bias and the corresponding curves are not shown in Fig. 2.

**Fig. 3:**
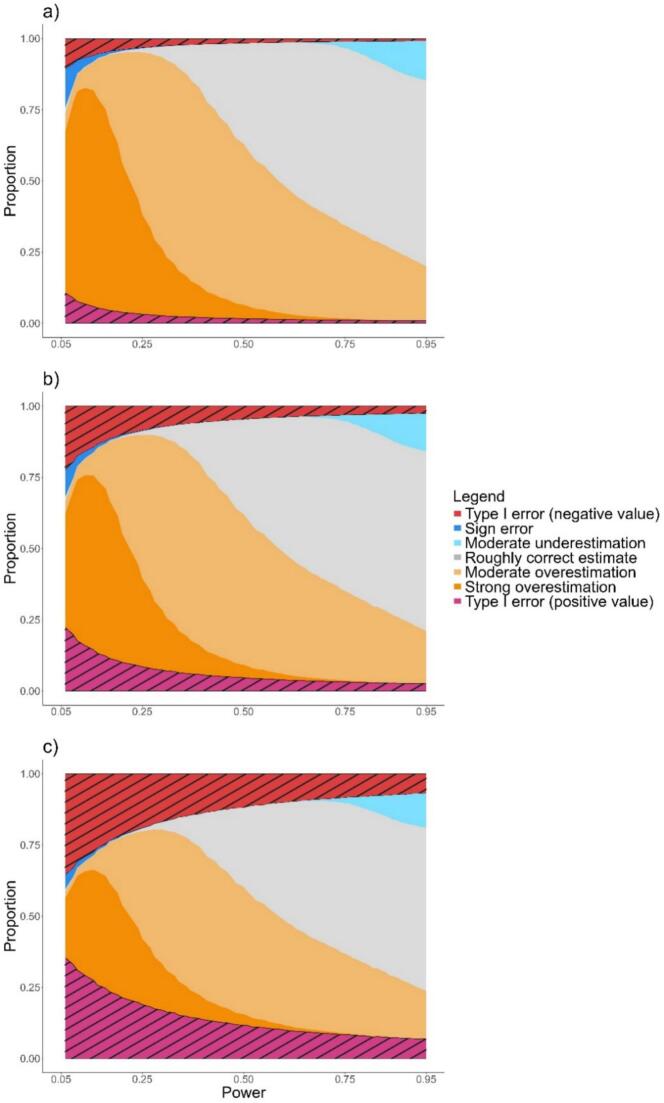
Proportions of estimation errors among statistically significant results as a function of power, under a proportion of tests with a true null effect of a) 25%, b) 50%, and c) 75%. For the other tests, the true effect was set to Cohen's d = 0.5. Areas corresponding to type 1 errors are hatched.

The consideration of a mixture of estimates of null and positive true mean differences allowed the consideration of type 1 errors among statistically significant results. Type 1 errors were symmetrical about the null value and increased in proportion as power decreased (Fig. 3). For any proportion of estimates of a true null mean difference (25, 50, 75%), below a power of 30 or 35%, the statistically significant results consisted almost entirely of overestimation errors, sign errors, and type 1 errors. However, the distribution of these errors depends on the proportion of true null hypothesis, with a greater type 1 error rate with increasing with the frequency of null hypotheses. For a frequency of 25% (Fig. 3a) and a power close to 5%, the sign errors were as frequent as type 1 error with a negative estimate.

## Discussion

While analyses that have a high probability of detecting a clinically meaningful effect yield predominantly accurate estimates when the results are statistically significant, this is not the case of low-power analyses: when power is 30% or less, approximately correct statistically significant results are rare. The relative bias was further exacerbated when the effect size was low but was always very high for powers as low as 20%, even for an effect size of 0.8, with at least a 2-fold increased estimate. It is noteworthy that even at high levels of power (≥80%) only about 65% of statistically significant results yield an approximately accurate estimate of effectiveness (defined here as an estimate within plus/minus 25% of the true value). The remainder represent moderate overestimation, which predominates at power of 80%, or moderate underestimation, which becomes increasingly prevalent as power approaches 100%. To help researchers, a Shiny app is available to calculate with simulations the relative bias, the type S and M errors at the following web link: https://c-jaksic.shinyapps.io/small_power_bias/. The R function used in this app is shown in Appendix 5.

Whether the two populations of interest one wishes to compare have the same variance, or whether the samples are of the same size does not seem to affect in any significant way the relative bias, nor dos the statistical test in use (classical *t*-test, Welch's t-test, or z-test). However, using a one-sided instead of a two-sided test increases the relative bias. This occurs because, if the posited alternative hypothesis correctly assumes a positive difference, no negative estimate can be statistically significant. Consequently, this exclusion eliminates estimates that could mitigate the inflation of positive estimates, thereby increasing the overall bias. Finally, reducing the alpha threshold diminishes the relative bias provided that the statistical power is maintained. Indeed, to maintain a desired statistical power, lowering the alpha level requires an increase in the sample size. This larger sample size results in more stable estimates that are less likely to reach extreme values that would pull the bias toward higher values.

At a more technical level, previous studies investigated the exaggeration caused by the significance filter on the effect size [13], [16], [17] using the Type M error. Instead, the present research used the relative bias on mean difference to quantify this phenomenon. In addition, we assessed the relative bias under various conditions related to the sample and to the statistical tests. The type M error was found to be systematically higher than the relative bias, especially when the power was low, and more sensitive to the effect size. This metric is the mean of the absolute values of statistically significant standardized effect sizes relative to the true value. Unlike our relative bias measure, the type M error is on the standardized effect scale, which is less commonly interpreted in clinical research. In contrast, the relative bias directly reflects the extent of inaccuracies on the scale used to draw conclusions. As a result, we recommend to methodologists interested in this phenomenon to use the relative bias as a direct measure of the effect of statistical significance filtering on the expected estimate rather than the type M error.

At low power, results are either accurate but statistically non-significant, or statistically significant but inaccurate, whether exaggerated or of the wrong sign. In scientific research, a wealth of results is constantly being published. Each analysis being performed tests a null hypothesis, although the true effect is always unknown. When the true effect is null, the issue is the type 1 error rather than a relative bias. When only looking at statistically significant results from analyses under a mix of null and non-null true effects, the type 1 errors become increasingly prevalent at low levels of power. While high-powered analyses yield non negligible type 1 errors when the proportion of tests with a true null effect is high (75%), it is the low-powered analyses that tend to produce the most concerning outcomes whatever the proportion of tests with a null true effect. When this proportion is low (25%) and power is low, the concern is more about the overestimation than the type 1 error. This highlights the problematic nature of low-powered analyses, in a context when significance filtering is a reality, ultimately undermining the generation of reliable knowledge.

The simulation study we conducted examines the impact of significance filtering on effect estimates. The conditions we analyzed, however, remain theoretical. In practice, filtering results based on statistical significance is less extreme, as non-significant findings are also published. Moreover, the power calculation used in our simulation relies on the true values of the mean difference and standard deviation. In real settings, these parameters are unknown, and sample size calculations are typically based on the smallest clinically relevant difference rather than the assumed effect size. Finally, the true power of an analysis is always unknown. Despite these limitations, the results we present provide a useful indication of the order of magnitude of the bias introduced by the significance filter under various conditions, and they offer valuable insight to inform real-world decision-making. Altogether these results indicate that application of statistical significance testing (or any procedure which highlights estimates that exceed a certain threshold) to observations from a sample size associated with an priori low-power test should be avoided, or if encountered in publications, met with scepticism [18]. Since significant results are more likely to be published [11] and cited [19], treatment effects reported in published low-powered studies are more likely to be exaggeratedly large. This also contributes to the lack of reproducibility of much research [18], [20], [21], since a statistically significant result from one underpowered analysis is unlikely to be reproduced in the next. The low-power bias should also be kept in mind when considering systematic reviews [22]. Incorporating low-powered studies in meta-analyses improves the precision of the pooled estimate but, in case of publication bias, aggregating several underpowered studies will yield a biased pooled estimate.

**Fig. 4:**
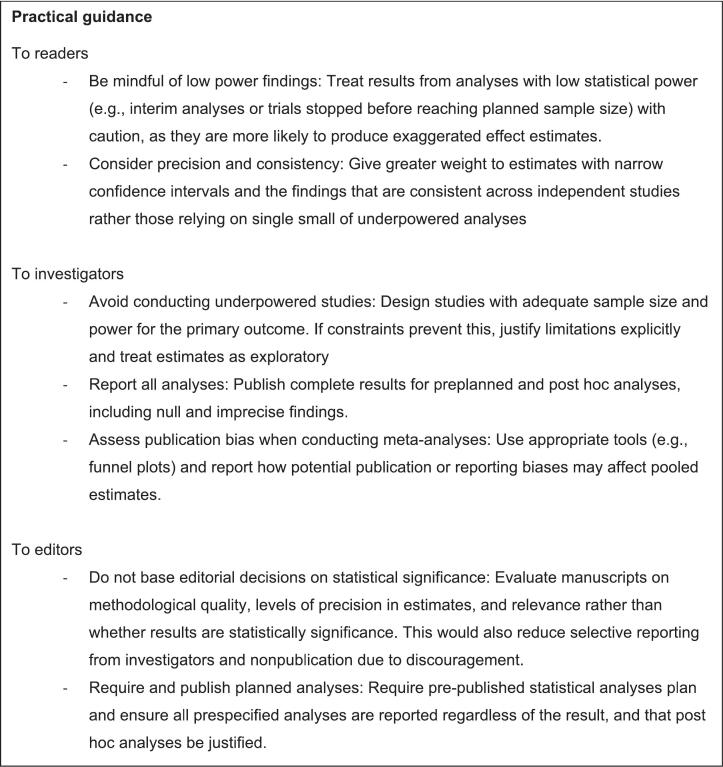
Practical guidance for readers of scientific publications, investigators and editors.

Below we introduce some recommendations to researchers, journal editors and readers of medical research articles. For a concise summary of our recommendations, see Fig. 4.

Reducing the statistical significance threshold well below 0.05 as commonly done in genome-wide association studies [23] or in interim analyses of clinical trials [24] only further decreases power and accentuates the resulting bias. For this reason, we would not recommend its use. Statistical procedures that shrink estimates toward the null value can help correct overestimation on average [14], but such procedures are currently rarely used. Our advice to researchers would be to resist temptation and outright avoid conducting low-power analyses. If low-power analyses were nonetheless performed, all results should be reported, regardless of statistical significance, and statistically significant findings should be accompanied by a caveat regarding possible over-estimation. In meta-analyses, researchers should investigate publication bias using appropriate methods and interpretate the results accordingly.

We recommend that journal editors not factor in statistical significance when deciding on accepting a paper for publication, and that they ensure that all low-power analyses that were planned are published, regardless of significance. Such an approach would also remove some obstacles to unbiased publication by being more welcoming of non-significant results, which are too often self-censured by discouraged researchers [25].

To readers of medical research articles, it is hard to identify whether one analysis is underpowered. Performing a power calculation for each analysis is theoretically possible but impracticable. But some identifiable situations are more at risk in clinical studies. In trials, although the power of the primary outcome analysis is most often controlled by the design, some contexts may lead to a lower power: early stop for efficacy since it is conducted only on part of the planned total sample size, failure to enroll the planned number of participants since a sample smaller than planned is analyzed, an event rate much lower than anticipated in the power calculation. For other analyses, the power is not controlled by the trial design. Subgroup analyses, which are restricted to a smaller sample, and analyses of safety events which are often infrequent, are likely underpowered. Regarding secondary outcomes tests, because no power calculation is typically conducted, the power can greatly vary across them. As the number of secondary outcomes increases, the risk for the readers to encounter underpowered analyses increases. For observational studies, the task is more difficult because the planned power is not always reported. Nevertheless, the sample size remains an important element to assess: a large cohort of several thousand patients is less prone to low power than a small study. Moreover, statistically significant differences that are very large (maybe too large to be true, to the reader's eye) should trigger a red flag. Any findings that are unusually ground-breaking, surprising, or that stand out from results published to date, are similarly suspect. In all cases, beware the authors' ability to articulate a coherent explanation for the finding post hoc. More generally, avoid distinguishing study results as notable or not, based on statistical significance.

## Contributors and sources

TP has proposed the analysis and wrote the first draft, CJ and CC performed the simulations and revised the paper. TP is the guarantor.

## Patient involvement

Patients or the public were not involved in this work.

## CRediT authorship contribution statement

**Cyril Jaksic:** Writing – review & editing, Writing – original draft, Methodology, Formal analysis, Conceptualization. **Thomas Perneger:** Writing – review & editing, Writing – original draft, Methodology, Formal analysis, Conceptualization. **Christophe Combescure:** Writing – review & editing, Writing – original draft, Methodology, Formal analysis, Conceptualization.

## Source of funding

None.

## Declaration of competing interest

The authors declare that they have no known competing financial interests or personal relationships that could have appeared to influence the work reported in this paper.

## Data Availability

No original data in the present study.

## Appendix A Supplementary data

Supplementary material Image 1
