# Supplementary material for: Statistically significant results from low-power analyses: A comedy of errors
Source: Glob Epidemiol. 2026 Jan 16;11:100250. doi: 10.1016/j.gloepi.2026.100250 (PMC12856849; doi:10.1016/j.gloepi.2026.100250)
Supplement: Supplementary file 1 — Supplementary material [file mmc1.docx]

Appendix 1. Formulae used to generate the simulated data and sample size calculation.

**Sample means**

Sample means were drawn from the following distribution:

$$\bar{x} \sim N\left( \bar{X}, \frac{\sigma}{\sqrt{n}} \right)$$

Where $\bar{X}$ is the population mean, $\sigma$ is the population standard deviation, and $n$ is the sample size.

**Sample variance**

Sample variances $s^{2}$ were drawn from the following distribution:

$$s^{2}\cdot\frac{n-1}{\sigma^{2}} \sim\chi^{2}\left( n-1 \right)$$

Where $\sigma^{2}$ is the population variance, $n$ is the sample size. For the simulations, values were randomly drawn from a chi-squared distribution of degrees of freedom $n-1$, and were then multiplied by the population variance and divided by $n-1$.

**Standard deviations for unequal variances groups**

To ensure that the Cohen’s d is equal to the mean difference, we set the standard deviations of both groups to 1. To simulate the condition of unequal variances, we set the standard deviations using the following formulae:

$$\sigma_{1}=\sqrt{\frac{2}{x^{2}+1}}$$

$$\sigma_{2}=x\sigma_{1}$$

Where $x$ (>1) is the factor by which $\sigma_{2}$ exceeds $\sigma_{1}$.

**Sample size calculation**

Sample size calculations were based on the formula for two independent means difference.

$$n_{1}=\left( 1+\frac{1}{m} \right)\frac{\left( Z_{1-\alpha/2}+Z_{1-\beta} \right)^{2}}{d^{2}}$$

$$n_{2}=n_{1}m$$

Where $n_{1}$ is the sample size in the group 1, $d$ is Cohen’s d, $m$ is the proportion of observations in group 1, $\alpha$ is the threshold alpha, and $1-\beta$ is the statistical power.

Appendix 2. Type S and M error, and the relative bias considered all significant results for different statistical power, with a true effect size of Cohen’s d = 0.2, and 0.8.

| **Cohen’s d** | **Power** | **Type S error rate (%)** | **Type M error** | **Relative bias (delta)** |
| --- | --- | --- | --- | --- |
| 0.2 | 0.1 | 3.9 | 3.64 | 3.22 |
|  | 0.2 | 0.5 | 2.27 | 2.22 |
|  | 0.3 | 0.1 | 1.81 | 1.80 |
|  | 0.4 | <0.1 | 1.57 | 1.56 |
|  | 0.5 | <0.1 | 1.41 | 1.41 |
|  | 0.6 | <0.1 | 1.29 | 1.29 |
|  | 0.7 | <0.1 | 1.20 | 1.20 |
|  | 0.8 | 0 | 1.13 | 1.13 |
|  | 0.9 | 0 | 1.06 | 1.06 |
|  | 0.95 | 0 | 1.03 | 1.03 |
| 0.8 | 0.1 | 7.5 | 11.16 | 2.13 |
|  | 0.2 | 1.0 | 3.04 | 2.09 |
|  | 0.3 | 0.1 | 2.01 | 1.73 |
|  | 0.4 | <0.1 | 1.65 | 1.51 |
|  | 0.5 | <0.1 | 1.45 | 1.38 |
|  | 0.6 | <0.1 | 1.33 | 1.28 |
|  | 0.7 | <0.1 | 1.23 | 1.19 |
|  | 0.8 | <0.1 | 1.15 | 1.13 |
|  | 0.9 | 0 | 1.08 | 1.06 |
|  | 0.95 | 0 | 1.04 | 1.03 |

Appendix 3. Relative bias of statistically significant deltas when only considering the positive or the negative deltas, as a function of power. In addition, the proportions of categories (moderate underestimation, roughly correct estimate, moderate overestimation and strong overestimation) of relative bias for positive deltas are reported. The results are shown for a Cohen’s d of 0.2 and 0.8.

|  |  |  | **positive deltas** | | | | |
| --- | --- | --- | --- | --- | --- | --- | --- |
| **Cohen’s d** | **Power** | **Relative bias**  **in negative deltas** | **Relative bias** | **Moderate**  **underestimation**  **(RB 0-0.75) %** | **Roughly**  **correct estimate**  **(RB 0.75-1.25) %** | **Moderate**  **overestimation**  **(RB 1.25-2) %** | **Strong**  **overestimation**  **(RB >2) %** |
| 0.2 | 0.1 | -3.17 | 3.48 | 0 | 0 | <0.1 | 100.0 |
|  | 0.2 | -1.96 | 2.24 | 0 | 0 | 34.1 | 65.9 |
|  | 0.3 | -1.51 | 1.80 | 0 | 0.2 | 74.9 | 24.9 |
|  | 0.4 | -1.29 | 1.56 | 0 | 16.0 | 73.1 | 10.9 |
|  | 0.5 | -1.10 | 1.41 | 0 | 37.6 | 57.4 | 5.0 |
|  | 0.6 | -0.99 | 1.29 | 0 | 51.5 | 46.2 | 2.3 |
|  | 0.7 | -0.86 | 1.20 | <0.1 | 61.7 | 37.3 | 1.0 |
|  | 0.8 | - | 1.13 | 5.1 | 64.6 | 30.0 | 0.3 |
|  | 0.9 | - | 1.06 | 12.0 | 64.8 | 23.2 | <0.1 |
|  | 0.95 | - | 1.03 | 14.1 | 66.5 | 19.4 | <0.1 |
| 0.8 | 0.1 | -1.58 | 2.43 | 2.0 | 7.5 | 24.7 | 65.8 |
|  | 0.2 | -1.48 | 2.13 | 0.2 | 4.6 | 38.3 | 57.0 |
|  | 0.3 | -1.24 | 1.74 | <0.1 | 9.7 | 66.3 | 23.8 |
|  | 0.4 | -1.07 | 1.52 | 0.1 | 23.0 | 67.5 | 9.4 |
|  | 0.5 | -0.96 | 1.38 | 0.3 | 39.1 | 56.5 | 4.1 |
|  | 0.6 | -0.83 | 1.28 | 0.6 | 51.0 | 46.3 | 2.0 |
|  | 0.7 | -0.71 | 1.19 | 2.0 | 60.1 | 37.1 | 0.8 |
|  | 0.8 | -0.78 | 1.13 | 5.4 | 64.3 | 30.0 | 0.3 |
|  | 0.9 | - | 1.06 | 11.4 | 65.3 | 23.3 | <0.1 |
|  | 0.95 | - | 1.03 | 13.8 | 67.0 | 19.2 | <0.1 |

Appendix 4. Scatterplot of all estimated mean differences (deltas) and Cohen’s d, in relation to the estimated pooled standard deviation. Different Cohen’s d values (0.2, 0.5, 0.8) were used with adapted sample sizes for a statistical power of 10%, with sample size per group of respectively n=24, n=4, n=2. Statistically significant estimates are shown in pink. The true value is represented by a dashed line.


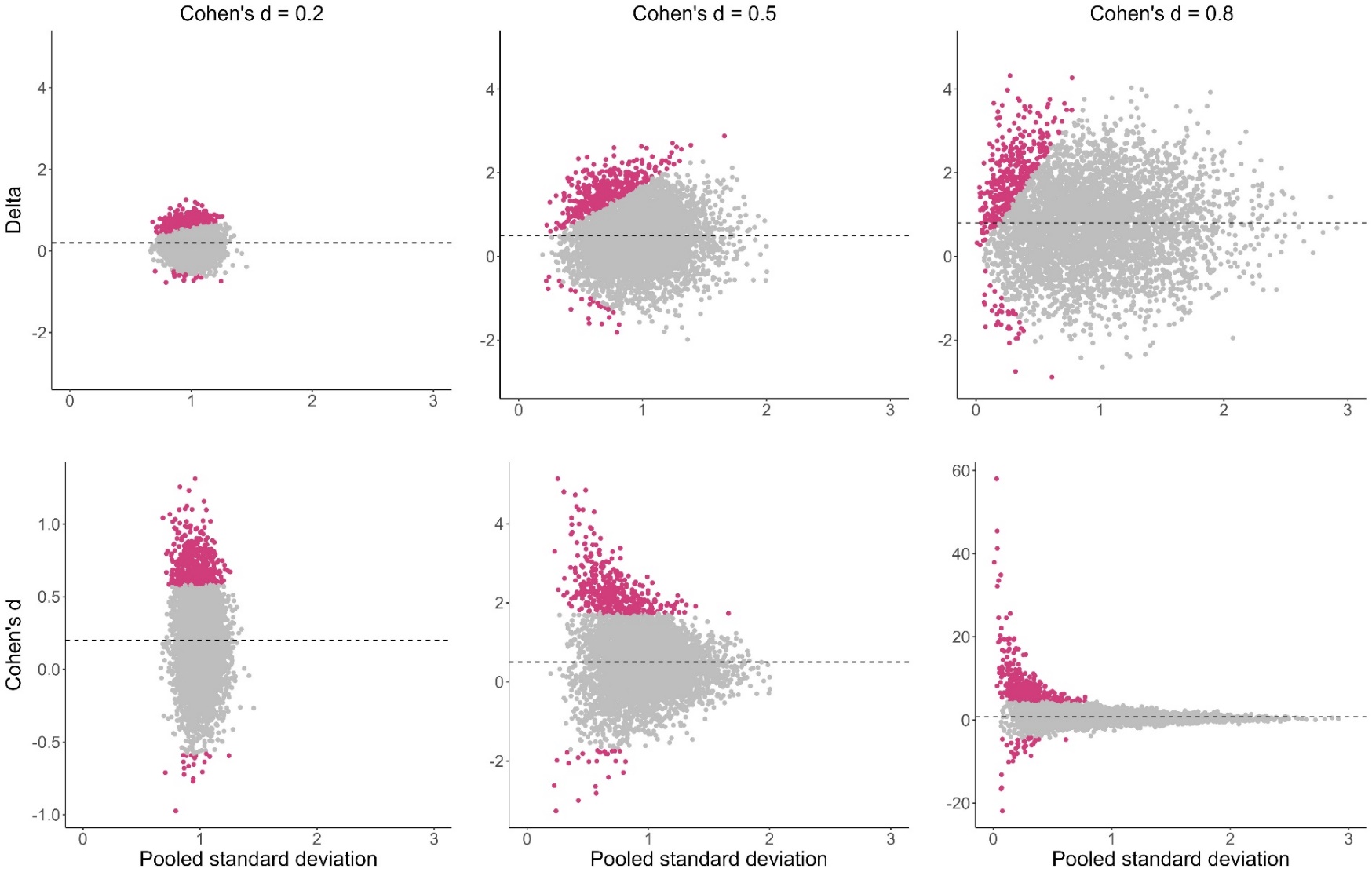


Appendix 5. The functions used to simulate the data.

### Function to draw sample means and standard deviations.

### For the formulae, see Appendix 1.

snorm = function(n, mean, sd, iterations){

means = rnorm(n = iterations, mean = mean, sd = sd/sqrt(n))

sds = sqrt(rchisq(n=iterations, df=n-1) * sd^2 / (n-1))

invisible(list(

means = means,

sds = sds

))

}

fct = function(powers = seq(.05, .95, by=.01),

alpha = .05,

alternative = c('two.sided', 'greater'),

test_statistic = c('t-student', 't-welch', 'z'),

delta = NULL,

sd1 = 1,

sd2 = NULL,

ratio_arm = 1,

proportion_of_H0 = 0,

n.sim = 500000,

seed = 123,

print_progress = FALSE){

set.seed(seed)

alternative = match.arg(alternative)

test_statistic = match.arg(test_statistic)

if (is.null(sd2)) sd2 = sd1

if (is.null(delta)) stop("'delta' must be specified (positive value)")

if (delta <= 0) stop("'delta' must be positive.")

if (proportion_of_H0<0 | proportion_of_H0>1) stop("'proportion_of_H0' must a probability value between 0 and 1.")

cohen_d_true = delta / sqrt((sd1^2 + sd2^2) / 2)

columns.names = c('theoretical_power', 'type_s_error_rate', 'type_m_error','type_m_error_only_pos','mean_delta_hat','mean_neg_delta_hat','mean_pos_delta_hat','mean_abs_delta_hat','relative_bias_d','relative_bias_delta','relative_bias_delta_pos','relative_bias_delta_neg','moderate_underestimation','roughly_correct','moderate_overestimation','strong_overestimation','actual_power','n.arm.1','n.arm.2','cohen_d','alternative','test_statistic','sd1','sd2','ratio_arm','n.sim','alpha','delta')

output = data.frame(matrix(ncol=length(columns.names), nrow=length(powers)))

names(output) = columns.names

row = 1

for (power in powers){

### Computes the required sample size

quantile = ifelse(alternative == 'two.sided', 1-(alpha/2), 1-alpha)

n.arm.1 = ceiling(((1 + 1/ratio_arm) * (qnorm(quantile) + qnorm(power))^2 ) / (cohen_d_true^2))

n.arm.2 = ceiling(n.arm.1*ratio_arm)

### Generate samples means and variances

arm1 = snorm(n=n.arm.1, mean=0, sd=sd1, iterations=ceiling(n.sim/power))

mean1 = arm1$means

var1 = arm1$sds^2

arm2 = snorm(n=n.arm.2, mean=delta, sd=sd2, iterations=ceiling(n.sim/power))

mean2 = arm2$means

var2 = arm2$sds^2

### Compute statistical significance

delta_hat = mean2 - mean1

pooled_sd = sqrt((var1*(n.arm.1-1) + var2*(n.arm.2-1)) / (n.arm.1+n.arm.2-2))

cohen_d = delta_hat / pooled_sd

if (test_statistic == 't-student'){

test.stat.value = delta_hat / (pooled_sd * sqrt(1/n.arm.1 + 1/n.arm.2))

df_t_student = n.arm.1 + n.arm.2 - 2

if (alternative == 'two.sided'){ p.value = (1-pt(abs(test.stat.value), df=df_t_student))*2

} else if (alternative == 'greater') p.value = 1-pt( test.stat.value, df=df_t_student)

} else if (test_statistic == 't-welch'){

test.stat.value = delta_hat / sqrt(var1/n.arm.1 + var2/n.arm.2)

df_t_welch = ((var1/n.arm.1 + var2/n.arm.2)^2) / ((var1/n.arm.1)^2/(n.arm.1-1) + (var2/n.arm.2)^2/(n.arm.2-1))

if (alternative == 'two.sided'){ p.value = (1-pt(abs(test.stat.value), df=df_t_welch))*2

} else if (alternative == 'greater') p.value = 1-pt( test.stat.value, df=df_t_welch)

} else if (test_statistic == 'z'){

test.stat.value = delta_hat / sqrt(var1/n.arm.1 + var2/n.arm.2)

if (alternative == 'two.sided'){ p.value = (1-pnorm(abs(test.stat.value), mean=0, sd=1))*2

} else if (alternative == 'greater') p.value = 1-pnorm( test.stat.value , mean=0, sd=1)

}

significant = p.value < alpha

### Select observations of interest

delta_signif = delta_hat[significant]

if (alternative == 'greater') delta_signif = delta_signif[delta_signif>0]

nb_delta_signif = length(delta_signif)

delta_signif_pos = delta_signif[delta_signif>0]

nb_delta_signif_pos = length(delta_signif_pos)

delta_signif_neg = delta_signif[delta_signif<0]

nb_delta_signif_neg = length(delta_signif_neg)

test.stat.value_signif = test.stat.value[significant]

test.stat.value_true = delta / sqrt(sd1^2/n.arm.1 + sd2^2/n.arm.2)

cohen_d_signif = cohen_d[significant]

### Populate the output dataframe

output$theoretical_power[row] = power

output$type_s_error_rate[row] = nb_delta_signif_neg / nb_delta_signif

output$type_m_error[row] = mean(abs(test.stat.value_signif)) / test.stat.value_true

output$type_m_error_only_pos[row] = mean(test.stat.value_signif[test.stat.value_signif>0]) / test.stat.value_true

output$relative_bias_d[row] = mean(abs(cohen_d_signif)) / cohen_d_true

output$mean_neg_delta_hat[row] = mean(delta_signif_neg)

output$mean_pos_delta_hat[row] = mean(delta_signif_pos)

output$mean_abs_delta_hat[row] = mean(abs(delta_signif))

output$mean_delta_hat[row] = mean(delta_signif)

relative_bias = delta_signif / delta

output$relative_bias_delta[row] = mean(relative_bias)

relative_bias_pos = delta_signif_pos / delta

output$relative_bias_delta_pos[row] = mean(relative_bias_pos)

relative_bias_neg = delta_signif_neg / delta

output$relative_bias_delta_neg[row] = mean(relative_bias_neg)

output$moderate_underestimation[row] = sum(relative_bias_pos<=.75) / length(relative_bias_pos)

output$roughly_correct[row] = sum(relative_bias_pos>.75 & relative_bias_pos<=1.25) / length(relative_bias_pos)

output$moderate_overestimation[row] = sum(relative_bias_pos>1.25 & relative_bias_pos<=2) / length(relative_bias_pos)

output$strong_overestimation[row] = sum(relative_bias_pos>2) / length(relative_bias_pos)

output$actual_power[row] = nb_delta_signif / length(delta_hat)

output$n.arm.1[row] = n.arm.1

output$n.arm.2[row] = n.arm.2

output$cohen_d[row] = mean(cohen_d)

output$alternative[row] = alternative

output$test_statistic[row] = test_statistic

output$sd1[row] = sd1

output$sd2[row] = sd2

output$ratio_sd[row] = sd1/sd2

output$ratio_arm[row] = ratio_arm

output$n.sim[row] = n.sim

output$alpha[row] = alpha

output$delta[row] = delta

if (print_progress) print(power)

row = row + 1

}

return(output)

}
